# Supplementary material for: Microbiome–Metabolomic Analysis Revealed the Immunoprotective Effects of the Extract of Vanilla planifolia Andrew (EVPA) on Immunosuppressed Mice
Source: Foods. 2024 Feb 26;13(5):701. doi: 10.3390/foods13050701 (PMC10930650; doi:10.3390/foods13050701)
Supplement: Supplementary file 1 [file foods-13-00701-s001.zip › foods-2883589-supplementary.pdf]

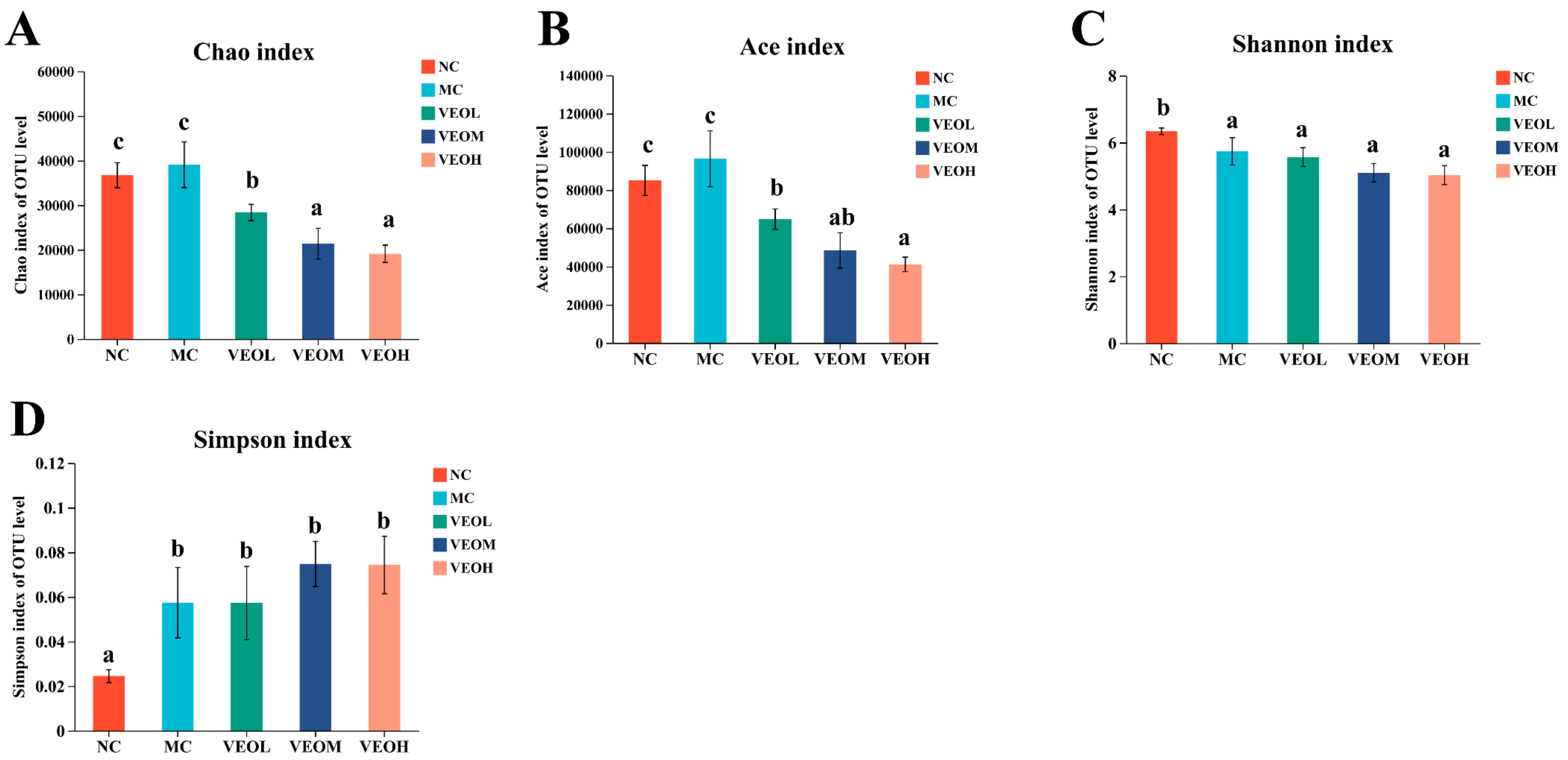

Figure S1. Effects of VOE on the α diversity of gut microbiota in immunosuppressed mice. (A) Chao 1, (B) ACE, (C) Shannon and (D) Simpson indexes. Values with different letters of the same indicator are significantly different ($p < 0.05$).

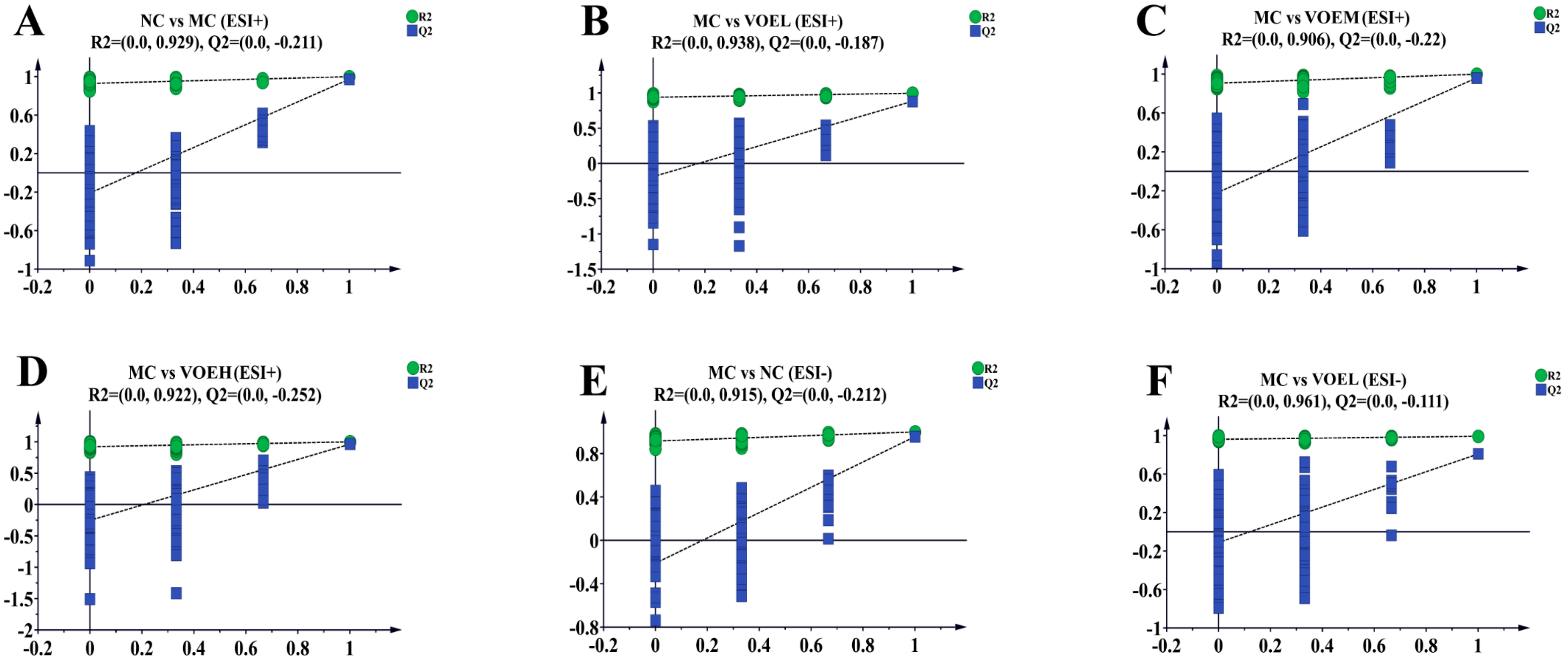
A
NC vs MC (ESI+)
R2=(0.0, 0.929), Q2=(0.0, -0.211)
B
MC vs VOEL (ESI+)
R2=(0.0, 0.938), Q2=(0.0, -0.187)
C
MC vs VOEM (ESI+)
R2=(0.0, 0.906), Q2=(0.0, -0.22)
D
MC vs VOEH (ESI+)
R2=(0.0, 0.922), Q2=(0.0, -0.252)
E
MC vs NC (ESI-)
R2=(0.0, 0.915), Q2=(0.0, -0.212)
F
MC vs VOEL (ESI-)
R2=(0.0, 0.961), Q2=(0.0, -0.111)
R2
Q2

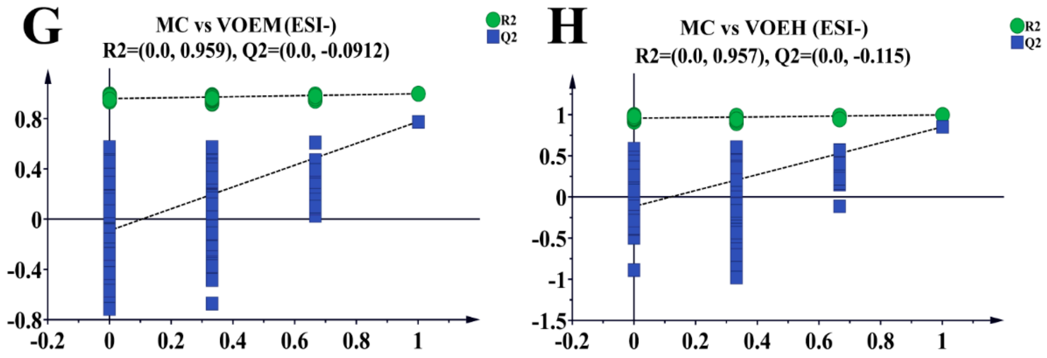

Figure S2. The permutation of OPLS-DA (A), (C), (E), (G) in positive ion mode and (B), (D), (F), (H) in negative ion mode in feces samples of mice

Table S1 Fecal differential metabolites in NC, MC, and VOEH groups

No.	Compound	MC vs NC		MC vs VOEH		HMDB ID/ KEGG ID	RT	Mass
		log_2FC	Change	log_2FC	Change			
1	7-Sulfocholic acid	2.27	up	2.27	up	HMDB0002421	9.70	488.24
2	3-Pyridylacetic acid	1.92	up	1.92	up	HMDB0001538	1.63	137.05
3	Cyclopassifloic acid B	2.31	up	1.49	up	HMDB0038388	21.12	580.40
4	Urobilinogen	-9.23	down	-8.85	down	C05791	10.74	590.31
5	Formamidopyrimidine nucleoside triphosphate	1.62	up	1.62	up	C05922	10.39	562.98
6	Ganglioside GD1a (d18:1/22:0)	1.94	up	1.94	up	HMDB0011799	10.59	1892.05
7	Cer (d18:1/18:0)	2.25	up	1.48	up	HMDB0000829	21.89	1092.73
8	L-655,240	2.85	up	3.52	up	HMDB0341170	25.10	373.13
9	N-arachidonoyl tyrosine	2.40	up	1.64	up	HMDB0062331	17.30	513.31
10	N-docosahexaenoyl phenylalanine	2.17	up	1.36	up	HMDB0062333	24.85	475.31
11	N-oleoyl tyrosine	2.46	up	1.07	up	HMDB0062337	24.27	445.32
12	PA (22:6/20:5)	1.93	up	1.15	up	HMDB0115418	9.38	766.46
13	PA (20:0/22:4)	1.95	up	1.22	up	HMDB0115085	22.32	824.59
14	Pantetheine	2.22	up	1.57	up	C00831	6.67	300.11
15	PC (15:0/0:0)	1.63	up	1.19	up	HMDB0010381	18.39	481.32
16	Rhizocticin A	1.96	up	1.16	up	C17944	4.89	397.13
17	Siroheme	1.96	up	1.96	up	C00748	10.82	914.22
18	Trichostatin A	2.22	up	1.44	up	HMDB0259177	9.41	302.16
